# Neutrophil-related signature characterizes immune landscape and predicts prognosis of esophageal squamous cell carcinoma

**DOI:** 10.1515/biol-2025-1210

**Published:** 2025-12-30

**Authors:** Zhipeng Hu, Zhi Ye, Yanan Guo, Miao Li

**Affiliations:** Department of Thoracic Surgery, Qinghai Provincial People’s Hospital, Xining 810000, Qinghai, China; Department of Oncology, Qinghai Provincial Tumor Hospital, Fifth People’s Hospital of Qinghai Province, Xining 810000, Qinghai, China

**Keywords:** esophageal squamous cell carcinoma, machine learning, prognosis, subtype, neutrophil

## Abstract

Esophageal squamous cell carcinoma (ESCC) is a globally prevalent malignancy, and neutrophils play a dual role in its progression and antitumor responses. This study aimed to investigate the prognostic significance of neutrophil-related genes (NRGs) and their potential in defining ESCC molecular subtypes. We identified differentially expressed genes (DEGs) and used univariate Cox analysis, followed by LASSO and multivariate Cox regression, to establish a five-gene prognostic signature. The model’s predictive performance was confirmed in an external validation cohort. To validate this, we performed qPCR to investigate the expression patterns of the five biomarkers, and the results were completely consistent with our data mining findings. Additionally, we performed consensus clustering to identify distinct molecular subtypes and assessed their characteristics through functional enrichment and immune microenvironment analyses. The prognostic model demonstrated robust predictive power. Patients with a high NRG score had significantly worse survival outcomes and a more complex immune microenvironment. Our analysis also revealed two distinct ESCC subtypes, Cluster 1 and Cluster 2, with Cluster 1 showing higher expression of immune checkpoints and significant differences in functional enrichment and immune microenvironment composition. In conclusion, NRGs serve as promising prognostic biomarkers in ESCC, providing insights into distinct molecular subtypes with clinical implications for personalized therapy. These findings underscore the need for further research on NRGs to advance our understanding of ESCC biology.

## 1 Introduction

Esophageal cancer (EC) is an aggressive malignant tumor with globally rising incidence and mortality rates [1]. It presents as two major subtypes: esophageal squamous cell carcinoma (ESCC) and esophageal adenocarcinoma (EAC) [2]. While ESCC is the most common type worldwide, particularly prevalent in East Africa and East Asia, EAC is more common in North America and Western Europe [3]. The primary risk factor for EAC is gastroesophageal reflux disease [4]. The etiology of EC is complex, involving multiple factors. Major risk factors for ESCC include smoking and heavy drinking, whereas EAC development is primarily associated with gastroesophageal reflux disease and obesity [5], 6]. Additionally, oral health and microbiota are also believed to play a significant role in its occurrence [7]. In terms of treatment, both early-stage endoscopic resection, later-stage surgical resection, and concurrent radiotherapy and chemotherapy have proven effective in improving survival for EC patients [8].

The relationship between cancer prognosis and immune cell infiltration has garnered significant attention in ESCC research. Tumor-infiltrating immune cells (TICs) play a crucial role in tumor progression and influence both prognosis and therapeutic outcomes [9]. In esophageal cancer, the expression patterns of immune-related genes can serve as predictors of prognosis and response to immunotherapy. For instance, TP53-related gene signatures have been demonstrated to predict immune cell infiltration, therapeutic response, and prognosis in patients with esophageal carcinoma [10]. Furthermore, immune gene-based prognostic models in esophageal cancer have been shown to effectively predict patient survival and can serve as potential biomarkers for immunotherapy [11], [12], [13], [14]. However, individual responses to cancer immunotherapy vary widely. Therefore, the identification of new and unique genes is expected to advance more targeted and effective treatment strategies, thereby providing a reference for the treatment of EC [15].

Within the tumor microenvironment (TME), a heterogeneous network of cancer cells, stromal elements and infiltrating immune populations – including macrophages, neutrophils, dendritic cells, natural killer cells and adaptive lymphocytes (T and B cells) – establishes a dynamic milieu that governs tumor initiation, progression and therapeutic response [16], 17]. Neutrophils are the most abundant phagocytic cells and can exert both protumorigenic and antitumorigenic functions [18]. Tumor-associated neutrophils have been shown to exhibit a range of antitumor activities [19], 20]. For example, neutrophils isolated from healthy individuals have been demonstrated to trigger autophagy pathways in breast cancer cell lines, leading to tumor cell death [21]. Neutrophil infiltration enhances the prognostic significance of CD8 + T cell infiltration in colorectal cancer and may effectively promote anti-tumor immunity [22]. Conversely, tumor-associated neutrophils can potentiate malignant progression by secreting pro-inflammatory chemokines such as interleukin-8 (IL-8) and potent angiogenic mediators including vascular endothelial growth factor (VEGF), thereby fostering neovascularization and tumor expansion [23]. Neutrophils additionally release proteolytic enzymes, notably matrix metalloproteinases, which degrade extracellular matrix components, thereby enabling enhanced tumour cell invasion and migration [24]. Neutrophils have both promotion and inhibitory effects in cancer, but the prognosis and immune landscape of NRGs in ESCC have not been completely deciphered.

In this study, we aimed to decipher the neutrophil-mediated mechanisms underlying ESCC progression. By combining multivariate Cox regression with machine learning, we identified five prognostic genes. These genes were subsequently validated using receiver operating characteristic curve (ROC) analysis in independent cohorts. Furthermore, consensus clustering uncovered two ESCC subtypes with distinct molecular profiles, which were characterized by differential immune infiltration and checkpoint expression. Our work not only delineates a novel neutrophil-related signature for risk stratification but also provides actionable targets for immunotherapy, thereby offering a framework for precision oncology in ESCC management.

## 2 Materials and methods

### 2.1 Data acquisition and preprocessing

**Figure 1:**
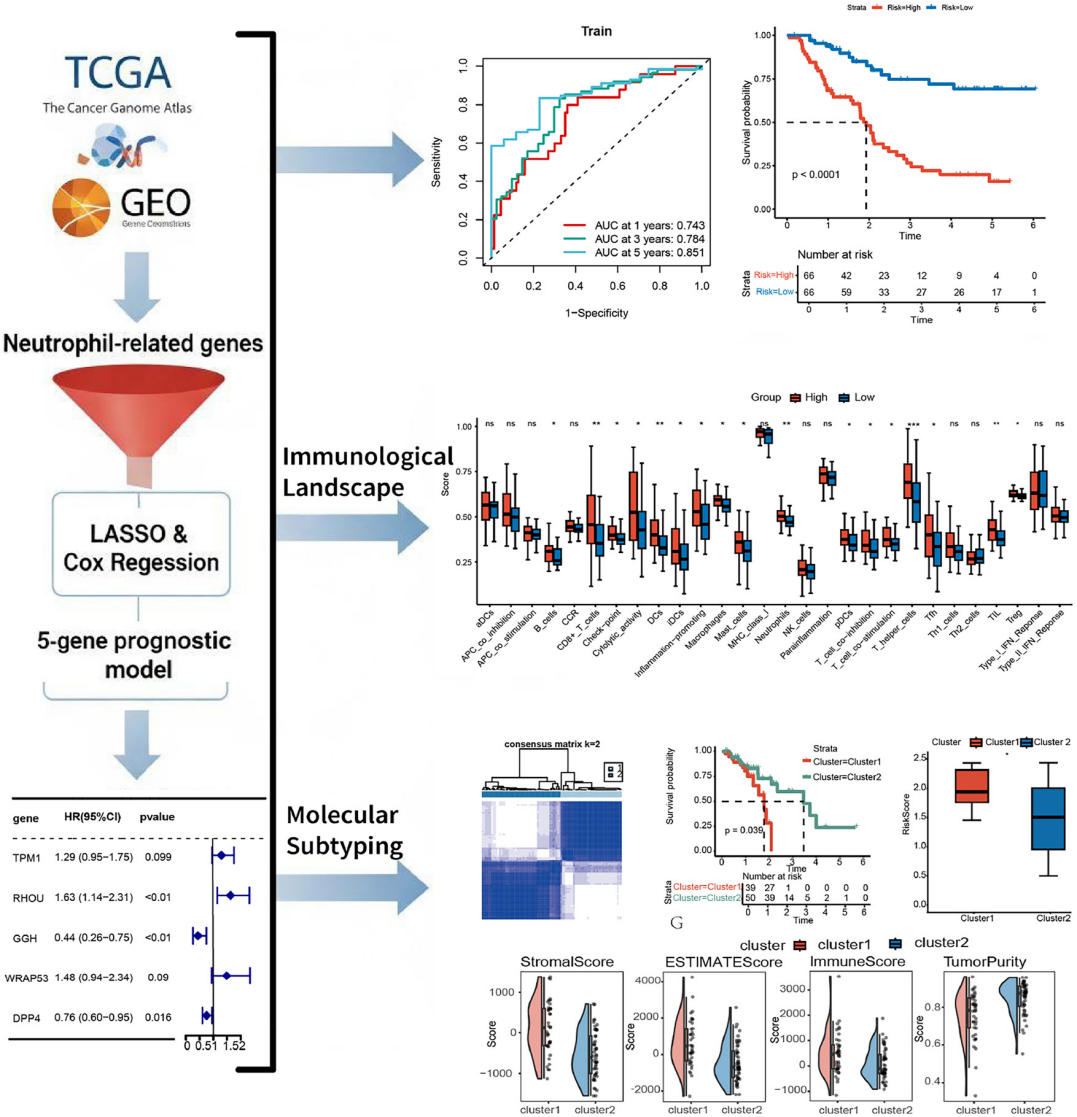
The complete workflow of this study.

Transcriptomic data and corresponding clinical information for ESCC were retrieved from UCSC Xena (https://xena.ucsc.edu/). To ensure data quality, patients with an overall survival (OS) shorter than 30 days were excluded from analysis. Repeated samples retain maximum expression size samples (*n* = 89). The 89 ESCC samples were further subjected to subsequent analyses. The GSE53625 dataset (*n* = 358, 179 tumors vs. 179 normal samples) and the GSE53624 dataset (*n* = 238, 119 tumors vs. 119 normal samples) were downloaded from the GEO database (https://www.ncbi.nlm.nih.gov/geo/). The GSE53625 dataset was merged with TCGA-ESCC data, and batch effects were removed. The combined dataset was randomly partitioned into two equal subsets, serving as a training set and a validation set. The GSE53624 dataset was used as an independent validation set. Neutrophil-related genes (NRGs) were obtained from MSigDB database, based on a previously published study [25]. The entire workflow is shown in Figure 1.

### 2.2 Differential expression and functional enrichment analysis

Principal component analysis (PCA) was performed to assess batch effects among the merged datasets. Differential expression analysis between tumor and normal tissues was conducted using limma package with Wilcoxon test applied to the GSE53625 dataset (|log2FC| > 0.585, FDR < 0.05). The differentially expressed genes (DEGs) were then intersected with the neutrophil-related genes (NRGs) to identify differentially expressed NRGs. To understand the biological processes and pathways associated with these genes, functional enrichment analyses for Gene Ontology (GO) and Kyoto Encyclopedia of Genes and Genomes (KEGG) pathways were performed using the ‘clusterProfiler’ R package. To assess the prognostic relevance of NRGs, univariate Cox proportional hazards regression was used to identify genes significantly correlated with patient survival outcomes (*p* < 0.05).

### 2.3 Prognostic model construction and validation

Survival-associated NRGs underwent LASSO regression with 10-fold cross-validation to prevent overfitting. Subsequent multivariate Cox regression established the final signature. Risk scores were calculated as:

$$\text{Risk score}=\sum \left({\text{Gene}\;\text{expression}}_{i}\times {\text{Coefficient}}_{i}\right)$$

Patients were divided into high-/low-risk groups using median cutoff. The characteristic genes were utilized for subsequent subtype classification. Feature gene expression patterns were visualized via boxplots and heatmaps.

### 2.4 Gene set enrichment analysis and pathway analysis

Gene Set Enrichment Analysis (GSEA v4.3.2) was performed to compare pathway activities between risk groups. Additionally, DEGs (|log2FC| > 0.585, FDR < 0.05) were subjected to GO/KEGG enrichment.

### 2.5 Independent prognostic evaluation

To evaluate the prognostic utility of the risk model, we used decision curve analysis (DCA) and time-dependent receiver operating characteristic (ROC) curve analysis, with the ‘ggDCA’ and ‘timeROC’ R packages, respectively. Kaplan–Meier survival curves were generated, and log-rank tests were conducted to compare OS between high- and low-risk groups, thereby evaluating the model’s discriminative capacity. Furthermore, a nomogram integrating the risk score with clinicopathological variables was constructed using the ‘rms’ and ‘survival’ R packages to facilitate individualized survival prediction. The predictive accuracy of the nomogram was assessed using calibration curves, which demonstrated a strong concordance between the predicted and observed survival outcomes.

### 2.6 Consensus clustering

Unsupervised clustering was performed on the expression profiles of the signature genes in the TCGA-ESCC samples using the ConsensusClusterPlus R package. This analysis successfully identified distinct molecular subtypes. To visualize the distribution and separation of these subtypes, PCA was applied, elucidating the underlying structure and heterogeneity among the identified clusters.

### 2.7 Immunological analysis

Immune cell infiltration was quantified using ssGSEA (for 29 immune signatures) and CIBERSORT. ESTIMATE scores, and Immune checkpoint expression were compared between risk groups and the subtype groups. These algorithms were employed to evaluate the correlation between the prognostic model and immune cell infiltration patterns within the TME. The ESTIMATE algorithm was used to assess the immune and stromal components of the TME, generating stromal scores, immune scores, and ESTIMATE scores. Higher scores are associated with a greater presence of the corresponding components, providing crucial insights into the tumor’s immunological landscape and its potential implications for patient prognosis.

### 2.8 Tumor mutational burden profiling and drug sensitivity analysis

Mutation profiles from TCGA-ESCC cohorts were analyzed to calculate the tumor mutational burden (TMB) scores for individual samples. The top 20 recurrently mutated genes in both high- and low-risk subgroups were statistically characterized. Mutation landscape visualization was performed using the ‘GenVisR’ R package to generate waterfall plots. To evaluate therapeutic responses across risk groups, the ‘pRRophetic’ R package was employed to estimate the half-maximal inhibitory concentration (IC50) of various chemotherapeutic agents. Lower IC50 values indicate higher drug sensitivity. Furthermore, the CellMiner database (https://discover.nci.nih.gov/cellminer/) was utilized to identify compounds whose sensitivity profiles significantly correlate with the expression of signature genes.

### 2.9 Quantitative reverse transcription polymerase chain reaction (qRT-PCR)

Both human esophageal epithelial cells (HET-1A) and esophageal squamous cell carcinoma cells (TE-1) were cultured in RPMI 1640 medium supplemented with 10 % fetal bovine serum (FBS). Total RNA was extracted from tissues or cultured cell lines using the Total RNA Kit (Yeasen, Shanghai, China), following the manufacturer’s protocol. Complementary DNA (cDNA) was synthesized from 1 μg of total RNA using the Hifiar III 1st Strand cDNA Synthesis SuperMix for qPCR (with genomic DNA digestion) and the cDNA Synthesis Kit (Yeasen). For quantitative PCR (qPCR), Hieff UNICON Universal Blue qPCR SYBR Master Mix (Yeasen) was used. Data were normalized to the expression of the reference gene, glyceraldehyde-3-phosphate dehydrogenase (GAPDH), to control for variations in expression levels. All experiments were independently repeated three times. The sequences of the primers used are listed in Table 1.

**Table 1:** Sequences of primers for qRT-PCR.

Gene name	Primer sequences (5′-3′)
TPM1	Forward: GGCATCCTGAGAGAAGTCCC
	Reverse: TGTTAGGGGCGCTCTCTTCT
RHOU	Forward: TCAATGACATTTCACACACGTCAG
	Reverse: GTAGACTGCCAGTGTCTGGTC
GGH	Forward: GGAGCCTCTCCGTGAAGAATTT
	Reverse: GGAGCCTCTCCGTGAAGAATTT
WRAP53	Forward: CACTGCCGGACAGGATGAA
	Reverse: TTTGGGACAGTGGCATCGAA
DPP4	Forward: TCTGCTGAACAAAGGCAATGA
	Reverse: AGTGTAAGTTTTGCGACTGTCA

### 2.10 Statistical analysis

All statistical analyses were conducted using R (version 4.4.2). Comparisons of continuous variables between groups were performed using the Wilcoxon rank-sum test. Survival outcomes were assessed via Kaplan–Meier estimators, with differences evaluated by the log-rank test. Cox regression models were employed to estimate hazard ratios (HRs) and corresponding 95 % confidence intervals (CIs). Time-dependent ROC curves were generated to evaluate the prognostic performance of the model at 1, 3, and 5 years. To control for multiple comparisons, *p*-values were adjusted using the Benjamini–Hochberg method. Data visualization was carried out using the ‘ggplot2’ and ‘ComplexHeatmap’ R packages. A *p*-value of <0.05 was considered statistically significant.

## 3 Results

### 3.1 Construction of a prognostic model based on the NRGs score in ESCC

**Figure 2:**
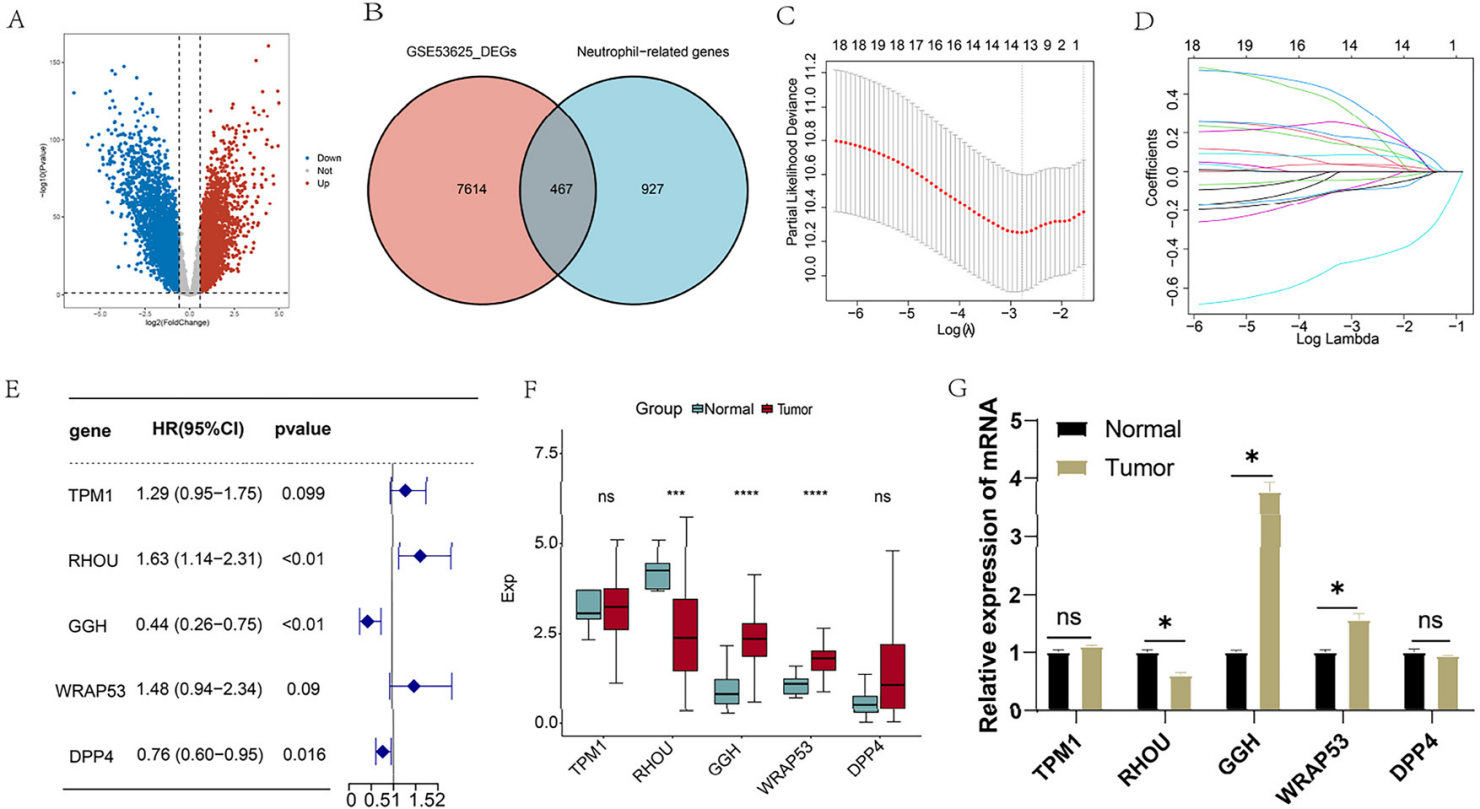
NRGs-DEGs identification. (A) Volcano plot displaying DEGs between tumor and normal tissues (GSE53625). (B) Venn diagram identifying overlap between DEGs and NRGs. (C) feature selection using LASSO regression analysis on candidate NRGs. (D) Coefficient profiles from LASSO regression of the five prognostic NRGs. (E) Multivariate Cox regression results for the 5 NRG signature. (F) The difference in expression of the five NRGs between normal and tumor tissues in TCGA dataset. (G) qRT-PCR analysis of TPM1, RHOU, GGH, WRAP53, and DPP4 expression in HET-1A and TE-1 cells. *p < 0.05, **p < 0.01, ***p < 0.001, ****p < 0.0001.

Differential expression analysis of tumor and normal samples from the GSE53625 database revealed significant alterations in gene expression profiles in ESCC. Specifically, a total of 8,081 DEGs were identified, with 4,095 genes downregulated and 3,986 genes upregulated (Figure 2A). Furthermore, we identified 467 genes common to both the neutrophil-related genes and DEGs (Figure 2B).

To assess the prognostic relevance of NRGs in ESCC, we first performed a univariate Cox regression analysis on 467 differentially expressed NRGs, identifying 19 genes significantly associated with overall survival (OS) (Supplementary Table S1). Subsequent we applied LASSO regression and multivariate Cox analysis to refined this list to five key prognostic genes: TPM1, RHOU, GGH, WRAP53, and DPP4 (Figure 2C–E). Based on these five critical NRGs (Supplementary Table S2), a risk model was constructed, with the risk score calculated as follows:

Riskscore = 0.2568*TPM1expressionvalue + 0.486*RHOUexpressionvalue – 0.8205*GGHexpressionvalue + 0.3936*WRAP53expressionvalue – 0.2789*DPP4expressionvalue

Notably, RHOU expression was reduced in ESCC tissues compared to normal counterparts, whereas GGH and WRAP53 were upregulated in tumor samples. These expression patterns suggest distinct roles for these genes in ESCC pathogenesis and underscore their potential as prognostic biomarkers (Figure 2F). To confirm the expression patterns of the five biomarkers, qRT-PCR was performed. The results show that the expression of the five genes is exactly consistent with data mining (Figure 2G).

### 3.2 Validation of the prognostic model

**Figure 3:**
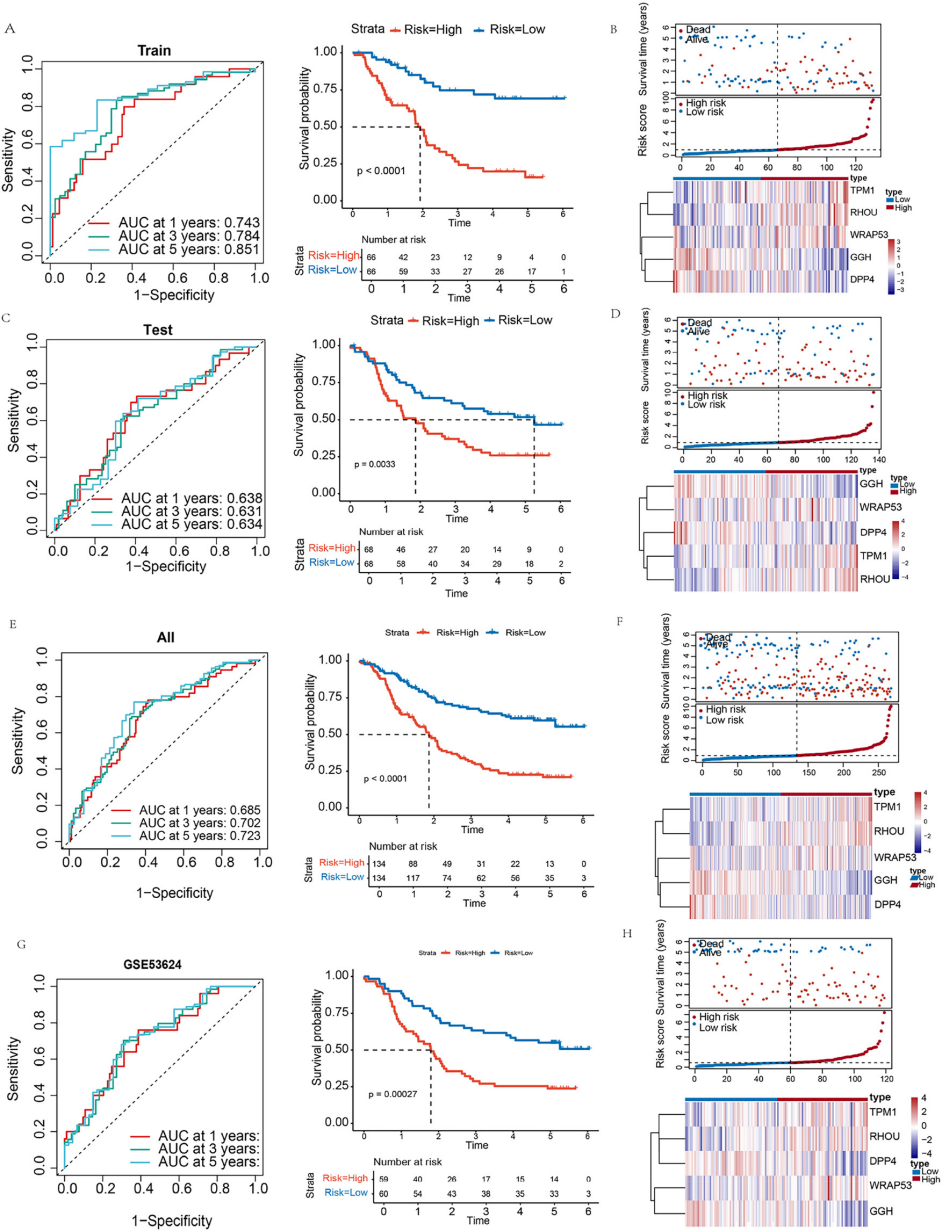
Prognostic model development and validation. (A) Training cohort: ROC analysis and survival stratification. (B) Training cohort: risk score distribution with corresponding survival outcomes and heatmap of gene expression. (C) Testing cohort: predictive performance and survival analysis. (D) Testing cohort: risk stratification and associated survival status and heatmap of gene expression. (E) Combined cohort: prognostic accuracy assessment and survival curves. (F) Combined cohort: risk score distribution with mortality correlation and heatmap of gene expression. (G) External validation (GSE53624): ROC and survival validation. (H) External validation (GSE53624): risk score distribution, survival status and heatmap of gene expression.

Patients were stratified into high- and low-risk groups based on the median risk score derived from prognostic model. In the training cohort, time-dependent ROC analysis yielded AUCs of 0.743, 0.784, and 0.851 for 1-, 3-, and 5-year survival, respectively. Kaplan–Meier analysis revealed significantly better OS in the low-risk group (Figure 3A). Risk score distribution and survival status plots further corroborated the prognostic value (Figure 3B). In the test cohort, the model maintained good predictive performance (AUCs > 0.60 across time points), and high-risk patients consistently exhibiting poorer outcomes (Figure 3C and D). Applying the model to combined datasets further confirmed its stability, with AUCs of 0.685, 0.702, and 0.723 for 1-, 3-, and 5-year survival. Similar survival trends and risk distributions were observed (Figure 3E and F). External validation in the GSE53624 cohort produced AUCs > 0.70, supporting the model’s robustness (Figure 3G and H). Consistent results were also obtained in the individual TCGA-ESCC and GSE53625 datasets, where AUCs exceeded 0.67 at all points. Notably, the 3- and 5-year AUCs in TCGA-ESCC surpassed 0.80 (Supplementary Figure 1), indicating strong long-term predictive capability.

### 3.3 Construction of nomogram

**Figure 4:**
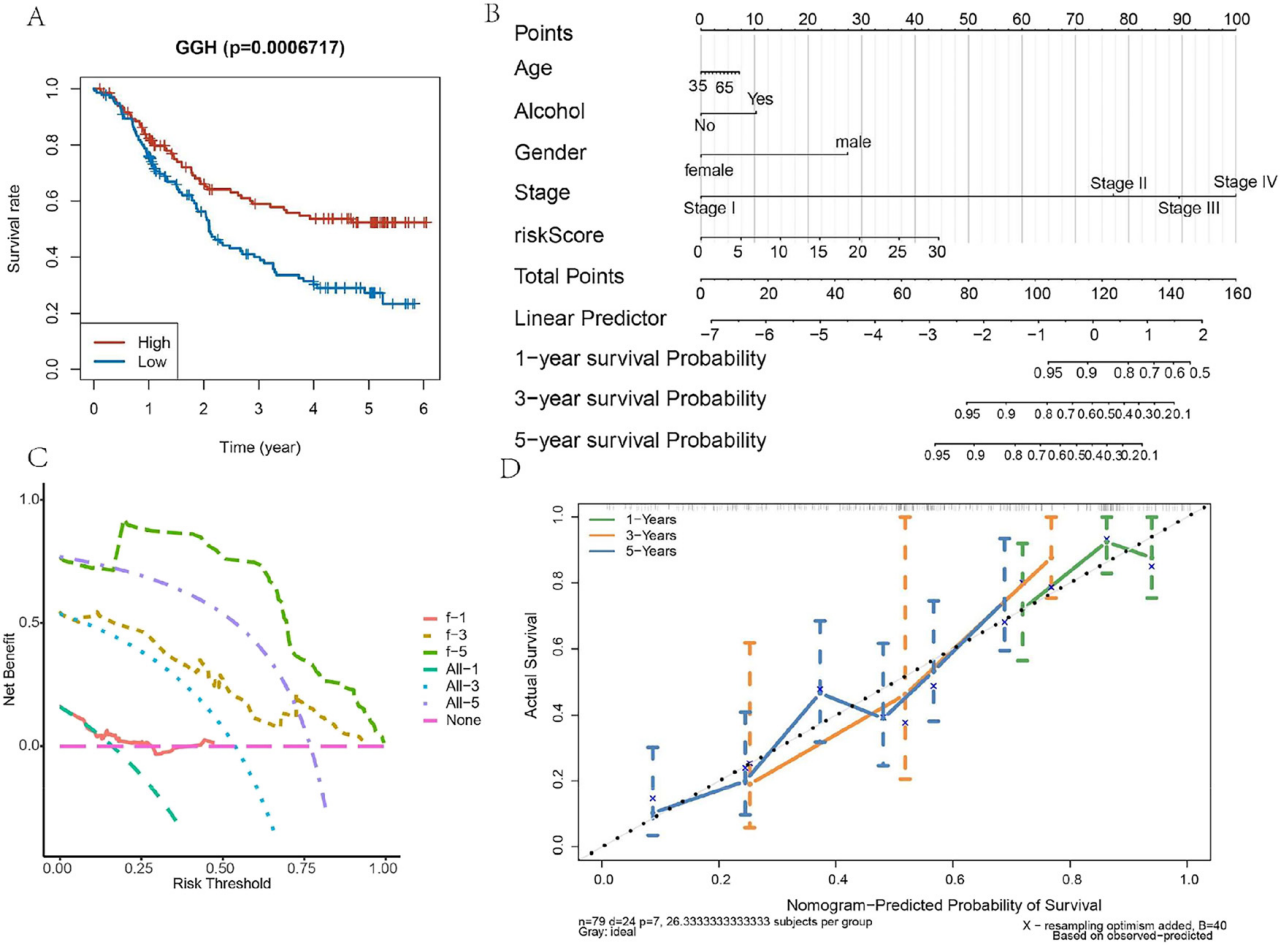
Independent prognostic factor analysis. (A) Kaplan–Meier curves for GGH genes. (B) Nomogram integrating risk score, alcohol, age, stage, and gender for survival prediction. (C) Decision curve analysis (DCA) demonstrating clinical net benefit. None (pink dashed line): Represents the strategy of treating no patients. All-1, All-3, All-5 (Cyan dashed line, blue dotted line, and purple dotted line): Represent the strategy of treating all patients, corresponding to 1-year, 3-year, and 5-year survival predictions. F-1, f-3, f-5 (orange-pink dashed line, yellow dotted line, and green dashed line): These lines represent the net benefit gained from using the prognostic model for decision-making, corresponding to 1-year, 3-year, and 5-year survival predictions. (D) Calibration curves aligning predicted versus observed survival rates. The colored lines (green, orange, blue) are the calibration curves for the nomogram’s predictions at different time points, representing the calibration curves for 1-year, 3-year, and 5-year survival predictions, respectively. The closer these colored lines are to the gray “ideal” line, the better the model’s calibration. The vertical bars on these lines represent the 95 % confidence intervals.

Kaplan–Meier survival analysis revealed that elevated expression of GGH was significantly associated with better OS in ESCC patients (Figure 4A). To facilitate individualized survival prediction, a prognostic nomogram was developed incorporating the risk score and relevant clinicopathological variables (Figure 4B). DCA for 1-, 3-, and 5-year survival indicated that the nomogram had strong predictive potential. Furthermore, calibration curves demonstrated a high concordance between predicted and observed outcomes, validating the model’s predictive accuracy (Figure 4C and D). Collectively, these results suggest that the risk score-based nomogram may serve as a potential prognostic instrument for prognostic assessment in clinical scenarios.

### 3.4 Function enrichment analysis

**Figure 5:**
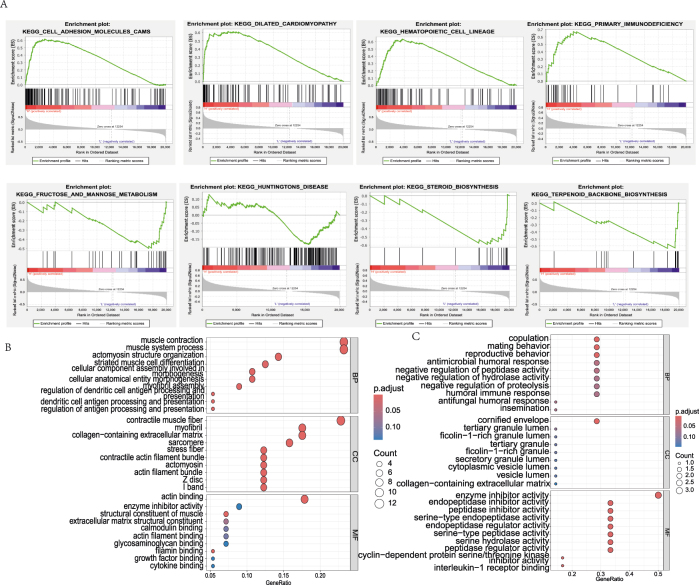
Enrichment analysis between high- and low-risk groups. (A) GSEA results of high- and low-risk groups. (B) GO enrichment of upregulated genes in the high-risk group. (C) GO enrichment of downregulated genes in the low-risk group. BP, biological process; CC, cellular component; MF, molecular function.

To investigate the biological functions and pathways associated with risk scores, we performed GSEA and GO enrichment analysis. The high-risk group showed significant enrichment in pathways such as Cell Adhesion Molecules (CAMs), dilated cardiomyopathy, hematopoietic cell lineage, and primary immunodeficiency (*p* < 0.05). In contrast, the low-risk group was enriched for pathways related to fructose and mannose metabolism, Huntington’s disease, steroid biosynthesis, and terpenoid backbone biosynthesis (Figure 5A). We performed GO analysis on DEGs in high and low risk groups. Upregulated genes in the high-risk group were predominantly involved in muscle-related processes, including muscle contraction, actomyosin structure organization, and muscle cell differentiation (Figure 5B). Conversely, the low-risk group exhibited enrichment of downregulated genes in copulation-associated functions, such as copulatory behavior, antimicrobial humoral response, and negative regulation of hydrolase activity (Figure 5C). These results reveal distinct molecular profiles between high- and low-risk ESCC patients. High-risk groups are linked to muscle contraction processes, while low-risk groups show copulation-related functions and potential immune regulatory mechanisms, suggesting prognostic heterogeneity and informing future therapeutic strategies.

### 3.5 Immunological landscape

**Figure 6:**
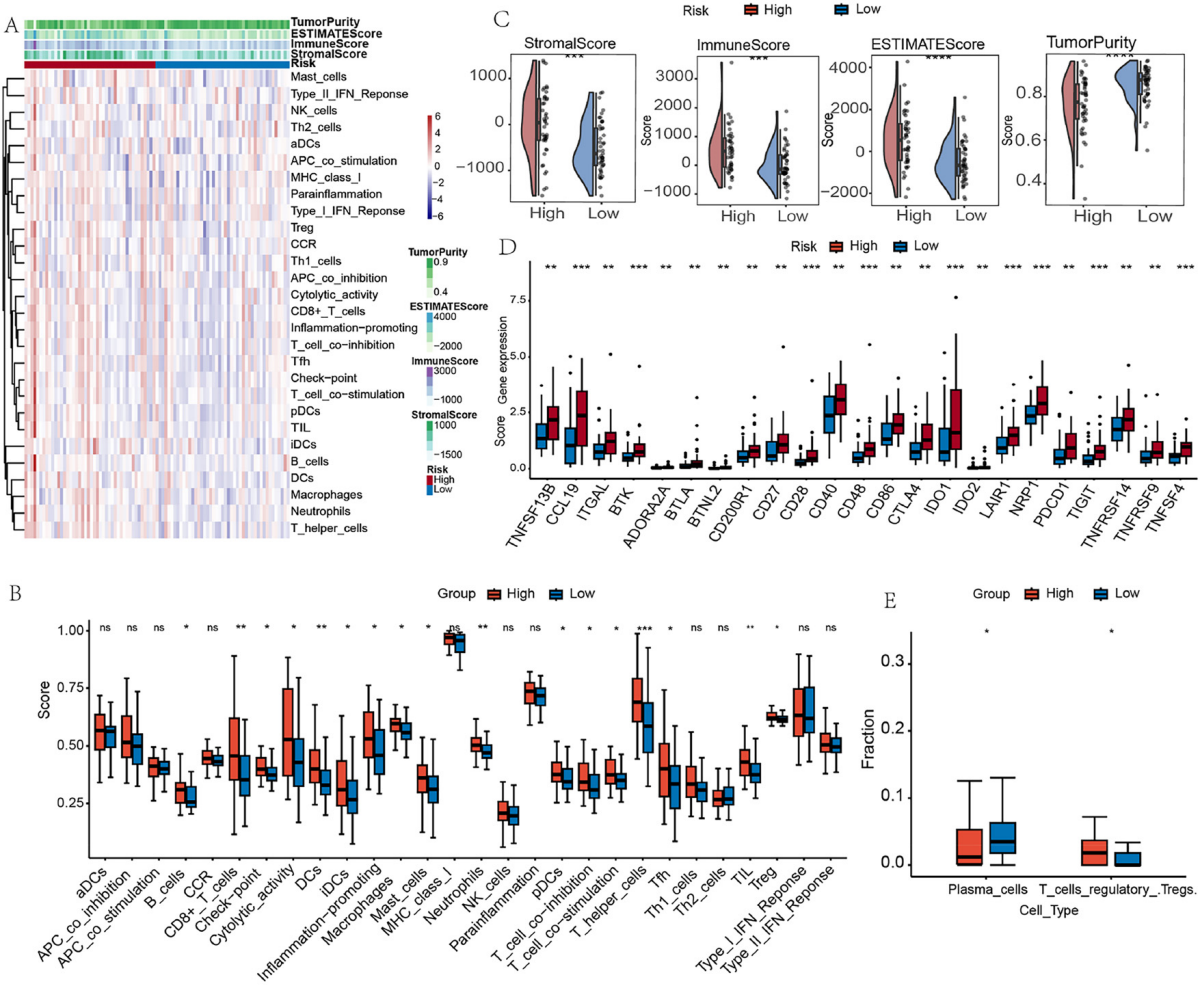
Comparative analysis of tumor immune microenvironment characteristics between ESCC risk subgroups. (A) Immune cell composition heatmap in ESCC TME. (B) Differential enrichment of immune-related pathways across risk groups. (C) ESTIMATE algorithm-derived stromal and immune scores comparison. (D) Risk-stratified expression patterns of immune checkpoint molecules. (E) CIBERSORT-quantified immune cell infiltration disparities. *p < 0.05; **p < 0.01; ***p < 0.001; ns, no significant.

The tumor immune microenvironment displayed risk-stratified characteristics in ESCC, as revealed by multi-platform analysis. ssGSEA quantification showed comprehensive immune activation in high-risk group, with significant elevation in adaptive immune markers (CD8+ T cells, B cells), antigen presentation components (DCs, iDCs), and immunosuppressive elements (checkpoint molecules, Tregs) (Figure 6A and B). Immune checkpoint profiling confirmed this immunosuppressive phenotype, while ESTIMATE algorithm demonstrated microenvironmental remodeling in high-risk tumors (Figure 6C and D). Furthermore, cellular deconvolution analysis revealed opposing infiltration patterns – plasma cell rich low-risk microenvironments versus Treg-dominated high-risk niches (Figure 6E), consistent with reported immune escape mechanisms [26].

### 3.6 Genomic and pharmacogenomic profiling

**Figure 7:**
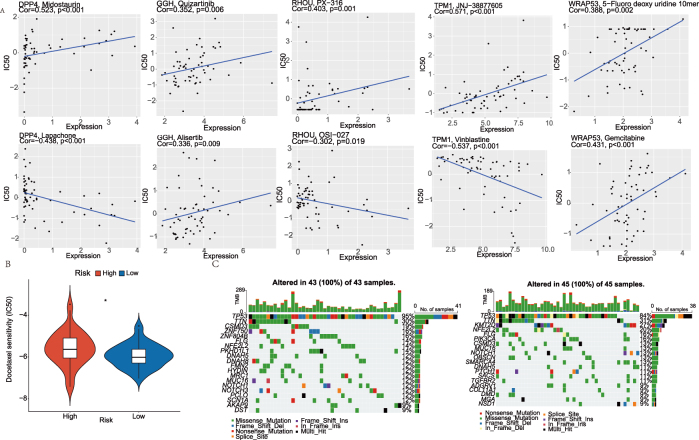
Drug sensitivity prediction and mutation in ESCC. (A) Correlation plot of drug sensitivity predictions using the CellMiner database. (B) Violin plot of IC50 values in high- and low-risk groups. (C) Waterfall diagram depicting contrasting mutation landscapes in high and low-risk populations. *p < 0.05.

Furthermore, using the CellMiner database, correlations between feature gene expression and drug sensitivity were predicted. Notably, DPP4 exhibited a strong positive correlation with Midostaurin (cor = 0.523) and strong negative correlations with Lapachone (cor = −0.438). In contrast, TPM1 showed a strong positive correlation with JNJ-38877605 (cor = 0.571) and strong negative correlations with vinblastine (cor = −0.537) (Figure 7A). Detailed results are provided in Supplementary Table S3. Drug sensitivity was assessed using the pRRophetic R package. The analysis showed that Docetaxel had lower IC50 values in the low-risk group (Figure 7B). TCGA mutation data were used to calculate the TMB in high- and low-risk groups. A waterfall plot depicting the top 20 genes by mutation burden was generated, which revealed that TP53 and TTN had a high mutation rate in both groups (Figure 7C).

### 3.7 Molecular subtyping based on signature genes

**Figure 8:**
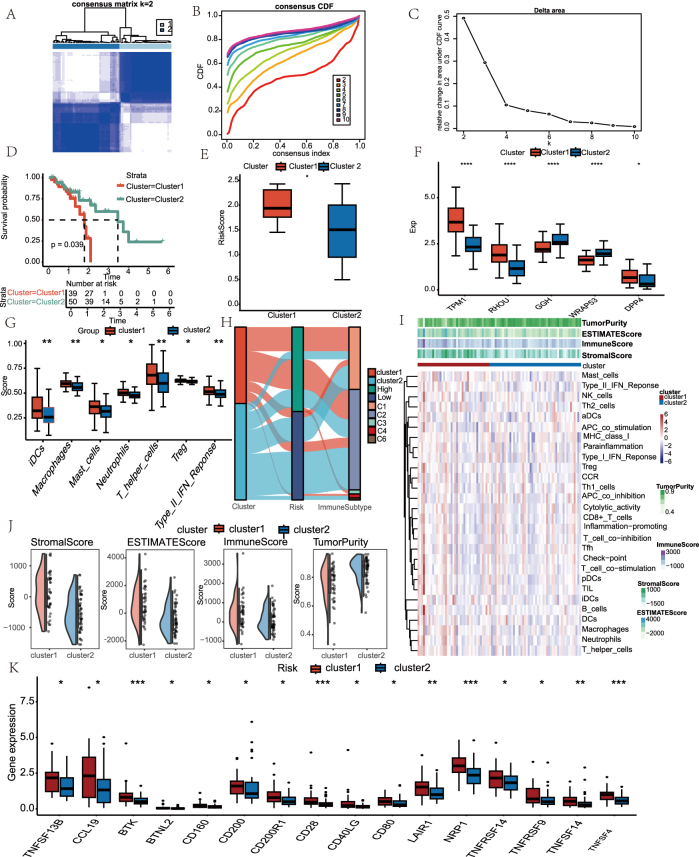
Molecular subtyping and immune correlates. (A) Consensus clustering analysis heatmap with two subgroups (k = 2). (B) Cluster stability assessment using cumulative distribution function (CDF). (C) Delta area curve determining optimal cluster number. (D) Kaplan–Meier survival curve among subtypes. (E) Boxplot of risk scores between subtypes. (F) Differential expression of signature genes across subtypes. (G) Boxplots displaying difference of immune cell infiltration profiles between clusters. (H) The interaction between immune subtypes and two risk groups was depicted as an alluvial diagram. (I) ssGSEA immune infiltration heatmap. (J) Boxplots of stromal immune, ESTIMATE scores and tumor purity among subtypes. (K) Comparison of differences in immune checkpoint expression between subtypes. *p < 0.05, **p < 0.01, ***p < 0.001, ****p < 0.0001.

Using TCGA-ESCC samples, consensus clustering based on model-derived feature genes identified two distinct molecular subtypes (Figure 8A), with CDF and delta area plots supporting optimal cluster separation (Figure 8B and C). Kaplan–Meier analysis revealed significantly poorer OS in Cluster 1 (Figure 8D), accompanied by higher risk scores compared to Cluster 2 (Figure 8E). Expression levels of the signature genes also differed markedly between the two subtypes (Figure 8F). CIBERSORT analysis uncovered distinct immune cell infiltration patterns across clusters, including differences in macrophages, mast cells, neutrophils, T helper cells, Tregs, immature dendritic cells (iDCs), and Type II IFN responses (Figure 8G). Integration analysis of molecular subtypes, risk scores, and immune subtypes revealed that Cluster 1 and the high-risk group were predominantly enriched in C1 (wound healing) and C2 (IFN-γ dominant) immune subtypes, both of which are associated with pro-tumor inflammation and immune activation. Conversely, Cluster 2 and the low-risk group were more frequently associated with C3 (inflammatory) and C4 (lymphocyte depleted), which are linked to immune suppression (Figure 8H). ssGSEA further highlighted differential immune infiltration, as shown in the heatmap (Figure 8I). ESTIMATE analysis demonstrated that Cluster 1 had significantly higher immune and stromal scores but lower tumor purity (Figure 8J). Finally, the expression levels of immune checkpoint genes differed significantly between subtypes, suggesting distinct immunoregulatory environments (Figure 8K).

## 4 Discussion

In 2024, EC ranked third among cancer-related deaths in China [27]. Therefore, identifying novel therapeutic targets and reliable prognostic markers is critical for improving ESCC treatment outcomes. Neutrophils, as a key subset in the tumor microenvironment, have profound effects on tumor immunity [28]. In this study, we developed a risk model based on NRGs, consisting of five characteristic genes, which demonstrates robust stability and independence in prognosis prediction. We found that high-risk patients not only have significantly reduced survival rates but also exhibit marked immune suppression in the tumor microenvironment. This is primarily manifested by significantly elevated levels of immune cells such as neutrophils and regulatory T cells (Tregs), along with an increase in the expression of immune checkpoint genes. Despite this high level of immune cell infiltration, high-risk patients have a worse prognosis, and this paradoxical phenomenon indicates a complex, immunosuppressive nature of their tumor microenvironment. This immunosuppressive state allows the tumor to evade host immune surveillance, which contributes significantly to disease progression. These findings provide valuable insights for developing personalized therapeutic strategies.

The five-gene prognostic signature derived from NRGs highlights their pivotal role in ESCC progression. Specifically, TPM1, RHOU, and WRAP53 function as risk factors, while GGH and DPP4 serve as protective factors. TPM1, a tropomyosin protein, is widely expressed and crucial forcell structure and function, influencing cellular motility and adhesion [29]. As a member of Rho GTPase family, RHOU participates in cytoskeletal reorganization, intercellular adhesion, cellular motility, intracellular transport, and proliferation control [30]. Numerous studies have confirmed the involvement of Rho GTPases in cancer development and progression [31], 32]. For example, adipogenesis signals have been shown to regulate the adhesion and migration of prostate cancer cells by modifying Rho GTPases [33], and lncRNA SNHG16 promotes ESCC development by interacting with EIF4A3 and regulating *RhoU* mRNA stability [34]. WRAP53 translates into a protein that associates with telomerase, facilitating the addition of telomeric repeats to chromosome ends, thus supporting the proliferation of stem and tumor cells [35]. Overexpression of WRAP53 in ESCC is associated with tumor progression and highlights its critical role in ESCC advancement [35]. GGH is a highly conserved lysosomal glycoprotein involved in folate metabolism [36]. GGH overexpression has been consistently identified across multiple malignancies, particularly in breast [37] and colorectal carcinomas [38], and pulmonary neuroendocrine tumors [37]. This elevated expression profile is significantly associated with aggressive tumor phenotypes and unfavorable prognosis [36], 39]. Conversely, DPP4, a cell surface glycoprotein, acts as a peptidase and regulates chemokine processing, signal transduction, and glucose metabolism. DPP4 is known to act as either an oncogene or tumor suppressor in different cancers. Its overexpression is associated with poor survival in squamous cell lung cancer [40], hepatocellular carcinoma [41], clear cell renal carcinoma [42], pancreatic cancer [43], and colorectal cancer [44], 45].

Interestingly, comprehensive immune analyses revealed significantly elevated levels of various immune cell types, immune cell infiltration scores, and immune checkpoint gene expression in the high-risk group, including neutrophils, regulatory T cells (Tregs), and BTK. Paradoxically, despite these markers generally indicating immune activation, the high-risk group was associated with poorer prognosis. This further confirms the high heterogeneity and complexity of the TME, indicating that despite active recruitment, immune cells may be unable to effectively perform their antitumor functions due to immune exclusion or suppression. The immunosuppressive capacity of Tregs allows tumors to circumvent host immune surveillance, contributing significantly to cancer immune evasion and disease progression [46]. Elevated neutrophil infiltration, particularly tumor-associated neutrophils (TANs), is increasingly recognized as a negative prognostic marker, as these cells can promote tumor growth, metastasis, and suppress effective immune responses [47]. Notably, high-risk patients also exhibited higher stromal, immune, and ESTIMATE scores compared to the low-risk group, suggesting a more infiltrated immune microenvironment. While tumor purity in the high-risk group was significantly lower than that in the low-risk group, indicating a more heterogeneous TME where immune cells are actively involved but potentially ineffective due to immune exclusion or suppression [48], 49].

In addition, our analysis revealed high-risk patients in our cohort demonstrated a higher sensitivity to Docetaxel, a chemotherapy agent commonly used in ESCC. Previous studies have demonstrated that immune scores serve as reliable prognostic markers and predictors of chemotherapy response [50], [51], [52]. For example, the immune microenvironment has been shown to predict outcomes and identify chemotherapy beneficiaries in gastric cancer [53]. In our analysis, the high-risk group exhibited elevated immune scores and increased expression of immune checkpoint molecules, suggesting that this subset may derive a potential survival benefit from immune checkpoint blockade [54], 55]. Our findings imply that ESCC patients in high-risk group might benefit from both Docetaxel and checkpoint inhibitor immunotherapy. Since chemotherapy not only directly kills tumor cells but also stimulates an effective anti-tumor immune response [56], 57], combining Docetaxel with an immune checkpoint inhibitor may offer an even greater survival benefit for the high-risk group. These results highlight the complex nature of the immune landscape in ESCC. Further studies are needed to understand the precise mechanisms by which the immune microenvironment modulates chemotherapy responses and to explore strategies that can enhance the effectiveness of chemotherapy and immunotherapy in high-risk patients.

Molecular subtyping based on neutrophil-related prognostic genes identified two distinct ESCC clusters with divergent immune landscapes. Cluster 1 exhibited heightened immune infiltration and elevated expression of immune checkpoint genes, yet was paradoxically associated with poorer survival, consistent with the high-risk neutrophil-related gene signature. This counterintuitive association between increased immune infiltration and adverse prognosis has been increasingly documented in cancer research, often attributed to immunosuppressive components within the tumor microenvironment (TME), such as regulatory T cells, M2 macrophages, and functionally impaired CD8+ T cells [58], 59]. Our comprehensive immunological and enrichment analyses further support this interpretation. Moreover, the pronounced upregulation of immune checkpoint genes in Cluster 1 suggests that this subtype may derive greater benefit from immune checkpoint blockade (ICB) therapy, providing a rationale for personalized immunotherapeutic strategies in ESCC. [60]. In summary, this integrative analysis linking neutrophil-associated gene expression with ESCC prognosis and immune subtypes yields new insights into intratumoral heterogeneity and highlights potential therapeutic vulnerabilities for precision immunotherapy in ESCC.

However, several limitations of this study should be noted. The moderate statistical significance of certain findings may reflect limited sample size and restricted availability of datasets, highlighting the need for validation in larger, independent cohorts. Furthermore, although several neutrophil-related genes appear potentially relevance to ESCC, their precise molecular mechanisms and functional interactions remain to be elucidated through more rigorous functional and prospective studies. For our future research, we will systematically validate how these core genes affect esophageal squamous cell carcinoma cell proliferation, invasion, and migration. We will also investigate their interaction with neutrophils and other immune cells by using methods such as gene knockdown, knockout, or overexpression.

## 5 Conclusions

This study not only expands theoretical understanding of neutrophil functions within the ESCC microenvironment but also provides a novel biomarker system for prognostic stratification and personalized therapy. By multi-dimensionally analyzing the interactions between gene signatures, immune microenvironments, and treatment responses, this study lays an important foundation for overcoming current challenges in ESCC immunotherapy resistance. Future research should further integrate experimental studies with clinical data to advance neutrophil-targeted therapies from mechanistic exploration to clinical practice.

## Supplementary Material

Supplementary Material

Supplementary Material
