# Supplementary material for: Neutrophil-related signature characterizes immune landscape and predicts prognosis of esophageal squamous cell carcinoma
Source: Open Life Sci. 2025 Dec 30;20(1):20251210. doi: 10.1515/biol-2025-1210 (PMC13011612; doi:10.1515/biol-2025-1210)
Supplement: Supplementary file 1 — Supplementary Material [file j_biol-2025-1210_suppl_001.docx]

**Neutrophil-related Signature Characterizes Immune Landscape and Predicts Prognosis of esophageal squamous cell carcinoma**

**supplementary information:**


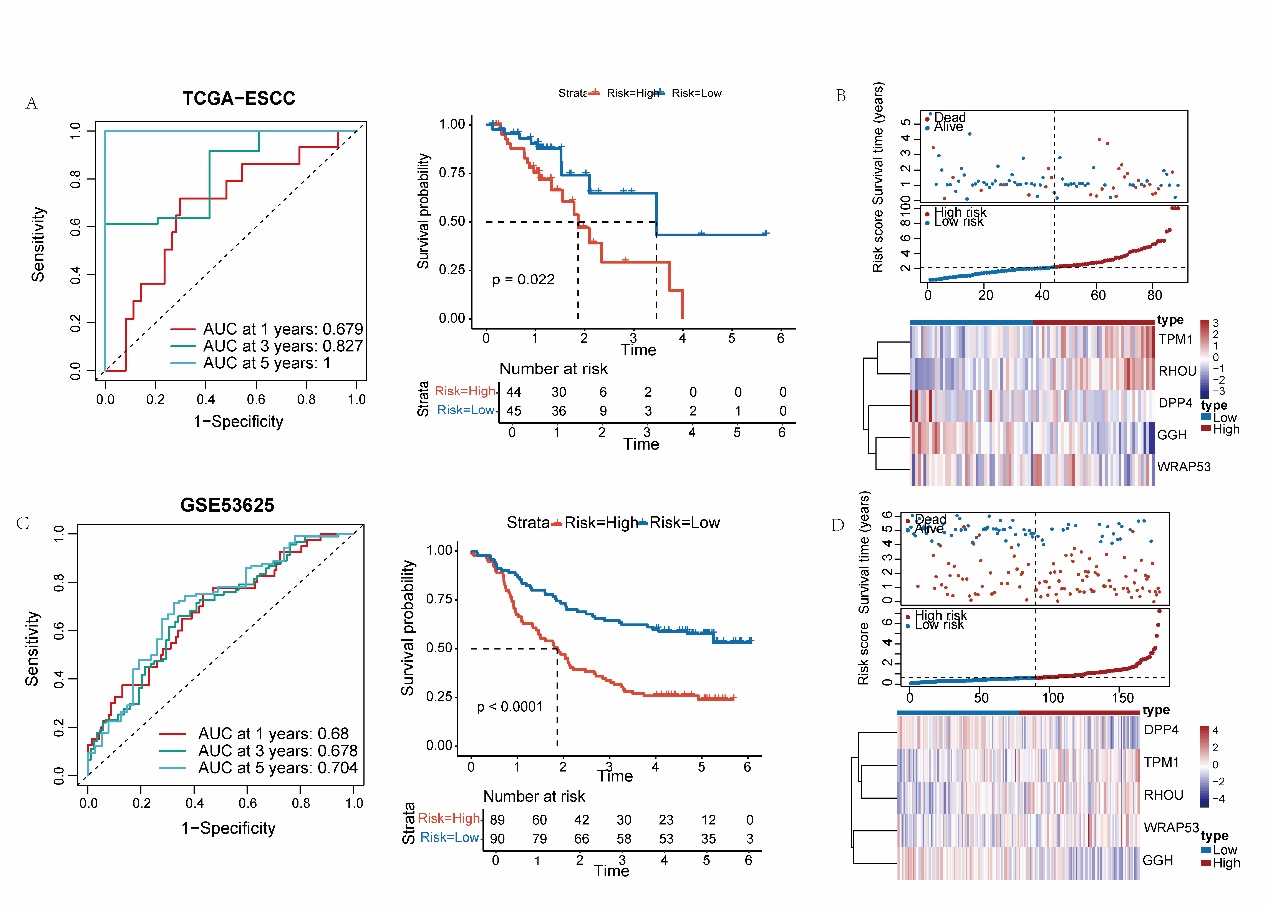


Supplementary Figure1. Validation of the prognostic risk model across TCGA-ESCC and GSE53625 cohorts.

**(A)** Time-dependent ROC curves for the TCGA-ESCC cohort at 1, 3, and 5 years demonstrate the predictive power of the model in estimating overall survival (B) Distribution of risk scores, survival status and heatmap of gene expression for TCGA-ESCC patients. **(C)** Time-dependent ROC curves in the GSE53625 validation cohort. **(D)** Risk score, survival distributions and heatmap of gene expression in the GSE53625 cohort
